# Integrating quantitative DCE-MRI parameters and radiomic features for improved IDH mutation prediction in gliomas

**DOI:** 10.3389/fonc.2025.1530144

**Published:** 2025-03-11

**Authors:** Meiping Ye, Zehong Cao, Zhengyang Zhu, Sixuan Chen, Jianan Zhou, Huiquan Yang, Xin Li, Qian Chen, Wei Luan, Ming Li, Chuanshuai Tian, Tianyang Sun, Feng Shi, Xin Zhang, Bing Zhang

**Affiliations:** ^1^ Department of Radiology, Nanjing Drum Tower Hospital, Affiliated Hospital of Medical School, Nanjing University, Nanjing, China; ^2^ Institute of Medical Imaging and Artificial Intelligence, Nanjing University, Nanjing, China; ^3^ Medical Imaging Center, Department of Radiology, Nanjing Drum Tower Hospital, Affiliated Hospital of Medical School, Nanjing University, Nanjing, China; ^4^ Department of Research and Development, United Imaging Intelligence, Shanghai, China; ^5^ Department of Radiology, Nanjing Drum Tower Hospital Clinical College of Nanjing Medical University, Nanjing, China

**Keywords:** glioma, dynamic contrast enhanced, quantitative parameter, radiomics, logistic regression

## Abstract

**Objectives:**

To develop and validate a multiparametric prognostic model, incorporating dynamic contrast-enhanced (DCE) quantitative parameters and multi-modality radiomic features, for the accurate identification of isocitrate dehydrogenase 1 (IDH1) mutation status from glioma patients.

**Methods:**

A total of 152 glioma patient data with confirmed IDH1 mutation status were retrospectively collected. A segmentation neural network was used to measure MRI quantitative parameters compared with the empirically oriented ROI selection. Radiomic features, extracted from conventional MR images (T1CE, T2W, and ADC), and DCE quantitative parameter images were combined with MRI quantitative parameters in our research to predict IDH1 mutation status. We constructed and analyzed Clinical Models 1–2 (corresponding to manual and automatic MRI quantitative parameters), Radiomic Feature Models 1–3 (corresponding to structural MRI, DCE, and multi-modality respectively), and a Multivariable Combined Model. We tried different usual classifiers and selected logistic regression according to AUC. Fivefold cross-validation was applied for validation.

**Results:**

The Multivariable Combined Model showed the best prediction performance (AUC, 0.915; 95% CI: 0.87, 0.96) in the validation cohort. The Multivariable Combined Model performed better than Clinical Model 1 and Radiomic Feature Model 1 (DeLong all p < 0.05), and Radiomic Feature Model 3 performed better than Radiomic Feature Model 1 (DeLong p < 0.05).

**Conclusions:**

Compared with the conventional MRI Radiomics and Clinical Models, the Multivariable Combined Model, mainly based on DCE quantitative parameters and multi-modality Radiomics features, is the most promising and deserves attention in the current study.

## Introduction

Glioma is the most common primary brain tumor, with its most frequent subtype glioblastoma, in particular, being one of the deadliest types of cancer. Brain tumors typically exhibit numerous genetic mutations, spanning several cellular pathways, that open multiple avenues to oncogenesis that no single intervention could conceivably block. The inclusion of mandatory molecular markers for diagnosis in the World Health Organization (WHO) Classification of Tumors of the Central Nervous System (CNS) was revised in 2021 (1), which has made a more rigid definition of prognostically distinct entities. Variations in glioma survival and response to therapy are ascribed to genetic and histological characteristics, particularly the degree of isocitrate dehydrogenase (IDH) mutation, the presence of 1p/19q co-deletion, and the tumor grade (2–4). IDH is the most critical prognostic marker, and the prognostic value of many other molecular markers (such as 1p/19q codeletion, TERT promoter mutation, and ATRX loss) depends on IDH (5), whose mutation is a positive prognostic factor. The product of the mutated IDH genes, d-2-hydroxyglutarate (D-2-HG), can induce global DNA hypermethylation and interfere with the immune system, thereby stimulating tumor growth (6). Previous studies have shown that the IDH1 status was the most prominent single prognostic factor (RR 2.7; 95% CI 1.6–4.5) followed by age, histological diagnosis, and MGMT (7). The survival benefit associated with surgical resection differs based on IDH1 genotype in malignant astrocytic gliomas. IDH1 mutant malignant astrocytomas are more amenable to surgical resection (8), and IDH1 mutation is associated with improved resection rates, progression-free survival, and overall survival (9). IDH inhibitors play a crucial role in the targeted therapy of gliomas and are one of the key types of drugs for glioma-targeted treatment. Selective inhibitors of mutant IDH, such as ivosidenib and vorasidenib, have been shown to reduce D-2-HG levels and induce cellular differentiation in preclinical models (10, 11). The phase III INDIGO trial has demonstrated that vorasidenib is superior to placebo in patients with non-enhancing grade 2 IDH-mutated gliomas after surgery (12). IDH mutations can affect tumor immunogenicity leading to the generation of neoantigens and changes in tumor-associated antigens (13). Second, they can shape the tumor microenvironment creating an immunosuppressive microenvironment and promoting tumor angiogenesis, which provides favorable conditions for tumor cells to escape immune surveillance (14). IDH mutations can influence immunotherapy targets through epigenetic regulation and can serve as biomarkers for predicting the efficacy of glioma immunotherapy (15).

The IDH genotype can be detected by surgery or biopsy, but it suffers from several drawbacks such as invasive operation, sampling error, tumor heterogeneities, and risk of surgical complications. Thus, it is essential to find a non-invasive technique. Conversely, magnetic resonance imaging (MRI), with its non-invasive, rapid, and extensive detection capabilities, and excellent resolution of soft tissue, is considered the most promising option to support clinical practice decisions (16). Numerous researches have investigated the possibility of using MRI-based Radiomics analysis to noninvasively facilitate the evaluation of prognosis, molecular subtyping, and tumor grading in gliomas (17–22). The pharmacokinetic parameters derived from dynamic contrast-enhanced magnetic resonance imaging (DCE-MRI) can be used to non-invasively predict the microvascular characteristics of tumor (23), and assess tumor characteristics and stage, providing independent prognostic indicators and enabling risk stratification for cancer patients (24, 25). Although DCE biomarkers have been validated through various reference methods and utilized for the assessment of a wide range of tumors, including gliomas (26), breast cancer (27, 28), and prostate cancer (29, 30), the process of manually segmenting DCE to extract image features is time consuming and prone to errors, and adversely impacts reproducibility, which is a significant issue in clinical applications, especially in longitudinal studies. The latest progresses in artificial intelligence (AI), especially deep learning, have demonstrated promising potential for dealing with these challenges (31, 32). Trained on extensive datasets of annotated MRI scans, deep learning models are capable of automating the segmentation process (33, 34). They not only bring about speed and high efficiency but also hold the prospect of minimizing human errors. However, integrating deep learning into clinical practice poses numerous technical challenges (such as the need for large, diverse training datasets and the management of imaging variability), as well as broader concerns regarding algorithm validation, integration into clinical workflows, and ethical considerations. To our knowledge, as of now, the research on predicting and evaluating IDH gene typing using a combination of DCE perfusion parameters and radiomic features through machine learning methods has not been explored in the literature. We hope to apply deep learning to mine and analyze glioma imaging data aiming to improve clinical decision making and patient care.

In this study, we proposed a fully automated method to predict IDH1 mutation in patients with glioma, requiring no user intervention, and proving highly suitable for clinical practice. We employed two methods, manual delineation of regions of interest and fully automated volume segmentation to extract apparent dispersion coefficient (ADC) and DCE perfusion parameters from the tumor core. The Radiomic features were extracted within the tumor core by the fully automated segmentation from DCE quantitative parameter images, ADC, T1CE, and T_2_W. Based on these features and parameters, we all constructed two clinical models, three Radiomic feature models, and a Multivariable Combined Model. We evaluated and compared the stability and predictive performance of these models for IDH genotyping and selected the most promising one.

## Materials and methods

This retrospective study was approved by the Research Ethics Committee of the Affiliated Nanjing Drum Tower Hospital of Nanjing University and performed in accordance with the Declaration of Helsinki.

### Patients

**Figure 1:**
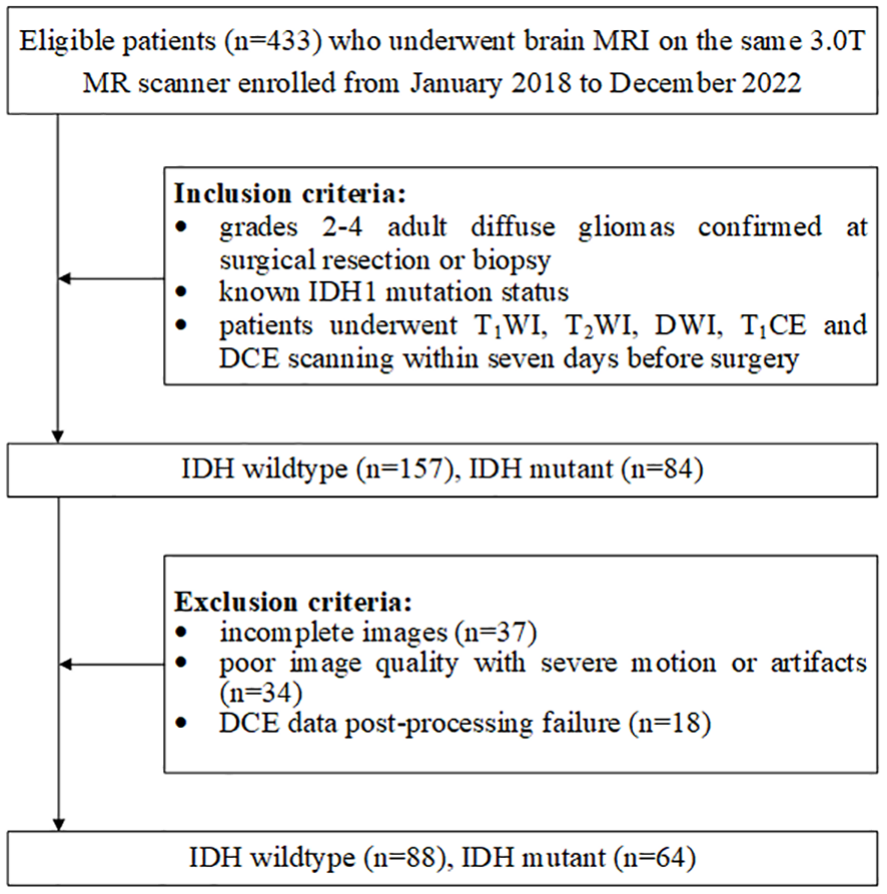
Flowchart of patient inclusion and exclusion.

Patients were retrospectively enrolled from our hospital between January 2018 and December 2022. The requirement for informed consent was waived due to the retrospective nature of this research. The patient enrollment process is shown in **Figure 1**. The exclusion criteria were as follows: (1) incomplete images, (2) poor image quality with severe motion or artifacts, and (3) DCE data post-processing failure. According to the above criteria, 152 patients [mean age, 56 years; age range, 22–76 years; 42 women (47.3%); IDH1 status (64 mutations and 88 wild type)] were recruited in this research.

### Image

MRI in the enrolled patients was performed on the same 3.0-T system (uMR790, United Imaging Healthcare, Shanghai, China) using a 32-channel phased-array head coil. All patients underwent the same MRI protocol including the following sequences: DWI, T_1_WI, T_2_WI, T_1_CE, and DCE. The DCE sequences consisted of T1 mapping and dynamic scan. For T1 mapping, variable flip-angle scans with flip angles FA 5°, 10°, 15° were acquired and used. Dynamic scan was a T1-gradient echo sequence with the following technical parameters: time of echo, 3.47 ms; time of repetition, 1.9 ms; flip angle, 13°; matrix size, 160 × 160; field of view, 240 × 220 mm^2^; slice thickness, 5 mm. A series of 1,800 images at 90 dynamic phases for 20 axial sections were obtained with a temporal resolution of 4 s for each dynamic phase. The contrast agent (Gadovist, 1 mmol/ml, Bayer Healthcare, Berlin, Germany) was administered (0.1 mmol/kg of bodyweight) through the antecubital vein via a power injector at a rate of 2 ml/s. The protocol details of the morphological sequences (DWI, T_1_WI, T_2_WI, and T_1_CE) are summarized in **Supplementary Table S1**. The quality of the sequences was assessed by two experienced neuroradiologists (Meiping Ye and SiXuan Chen), with more than 5 years of experience in neuro-oncological radiology.

### Tumor automatic segmentation

In this study, we used the VB-Net (35), a segmentation neural work, to obtain the region of interest (the tumor core) according to T_1_CE and T_2_WI. The VB-Net is a modified 3D convolutional neural network, which combines V-Net with innovative bottleneck structures and computes much faster than V-Net (36). VB-Net, which has been published, is an advanced adaptation of the segmentation-centric V-Net architecture. The improvements in VB-Net include the replacement of traditional convolutional layers in the down- and upsampling modules with a bottleneck structure. This bottleneck structure is designed to optimize parameter efficiency and is composed of three sequential convolutional layers: the first with a kernel size of 1 × 1 × 1, the second with 3 × 3 × 3, and the third reverting back to 1 × 1 × 1. This arrangement reduces redundancy and improves computational efficiency while maintaining performance integrity. Furthermore, VB-Net employs a cascaded framework tailored to accommodate varying regions of interest (ROIs) with multiple sizes. This adaptability ensures its suitability for diverse segmentation tasks providing robust and accurate results. For loss function optimization and enhanced model training, VB-Net integrates a combination of common Dice loss, pixel-class cross-entropy loss, and focal loss. This multi-faceted loss strategy ensures a well-constrained parameter update process contributing to the overall stability and performance of the network. As far as we know, the VB-Net has been successfully applied to the segmentation of multiple organs and lesions in previous researches (37–39).

**Figure 2:**
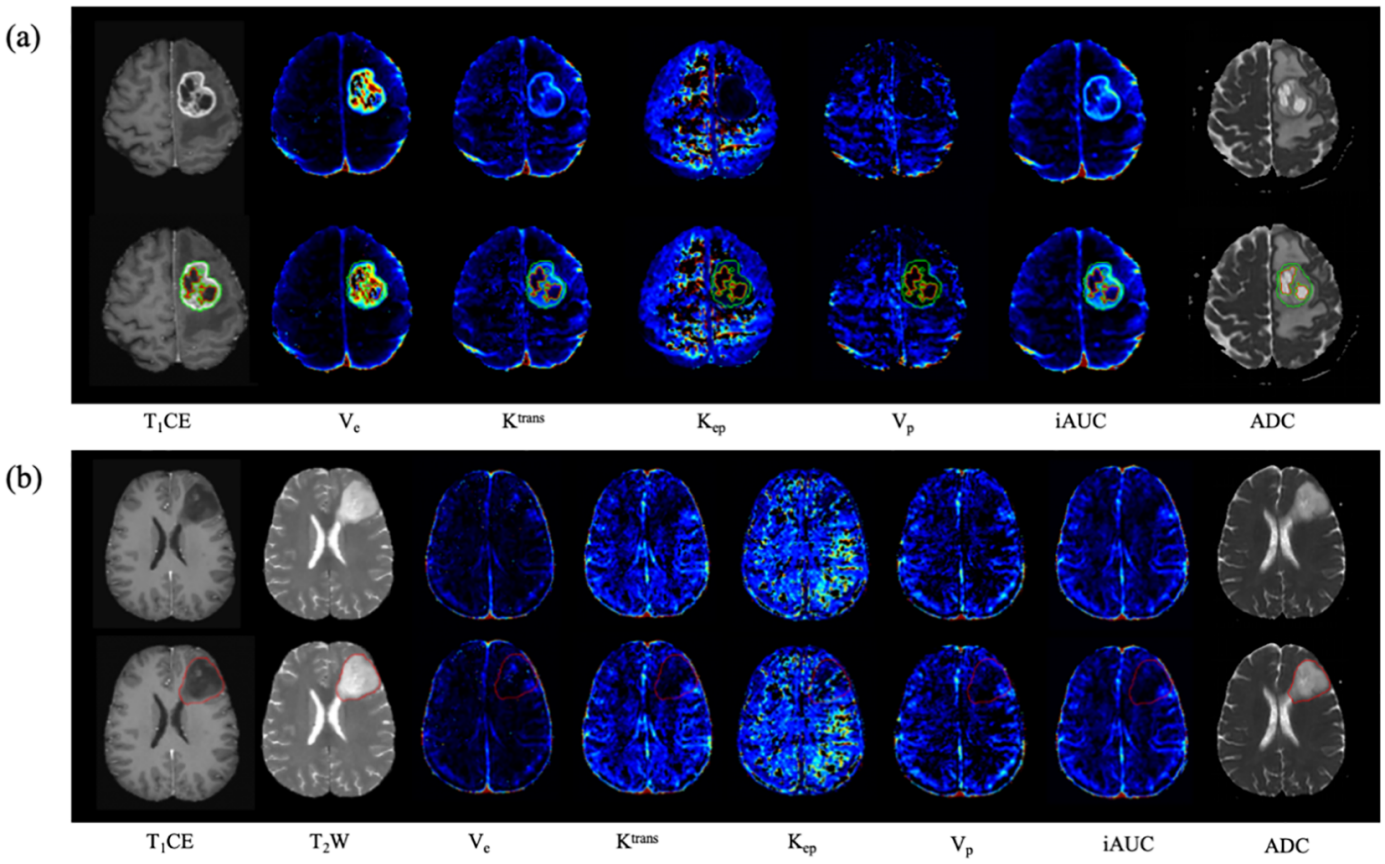
Examples of the automated segmentation and co‐registration with other sequences. **(a)** a case of glioblastoma. The lesion showed obvious enhancement, and tumor core was automatically segmented based on T1CE. **(b)** a case of diffuse astrocytoma (WHO 2 grade, IDH1-mut). The lesion showed no obvious enhancement, and tumor core was automatically segmented based on T_2_WI.

In the segmentation model construction phase, we exclusively used 1,000 cases (comprising T_1_CE and T_2_WI) from the public BraTs2021 dataset (http://www.braintumorsegmentation.org) for training and validation, and 250 cases were used to test the model’s performance. Additionally, we supplemented our analysis by incorporating a private dataset, which consisted of 204 T_1_CE images and 184 T_2_WI images. To construct the segmentation masks, we merged the labels from BraTS2021 based on the defined regions of interest, specifically targeting the whole tumor region in T_2_WI and the tumor core in T_1_CE. Notably, the thickness of all selected images was less than 1 mm ensuring high-resolution data for analysis. Regarding preprocessing, for the private dataset, we applied N4 bias field correction using the SimpleITK package. Subsequently, the T_1_CE images were spatially aligned with the T_2_WI image spacing using the ANTs tool. Finally, all image intensities were normalized to the range [−1, 1] enabling consistency across all input data. These preprocessing steps were critical in mitigating imaging artifacts and ensuring uniformity between datasets. Given the distinctions between the two segmentation tasks - whole tumor segmentation in T_2_WI and tumor core segmentation in T_1_CE - separate models were constructed for each. The segmentation models were evaluated using the Dice similarity coefficient (DSC). The DSC of the whole tumor segmentation model in BraTS2021 is 0.83 and 0.70 in the private dataset. Meanwhile, the DSC of the tumor core segmentation model in BraTS2021 is 0.72 and 0.85 in the private dataset. **Figure 2** shows examples of automated segmentation and co‐registration with other sequences.

### Region of interest analysis and quantitative MRI parameter acquirement

DCE-MRI data were imported into the United Imaging post-processing workstation for processing (United Imaging Healthcare, Shanghai, China). Arterial input functions were extracted in a manual manner, with the ROI positioned in the middle cerebral artery (40). Five quantitative pharmacokinetic DCE-MRI parameters (K^trans^, K_ep_, V_e_, V_p_, and iAUC) were acquired using the improved Tofts–Kermode two‐compartment model.

Two kinds of sketching methods of ROI, manual delineation and automatic segmentation, were applied to obtain regional-DCE parameters in our research. For the manual way, first, the quantitative MRI parameter maps (K^trans^, K_ep_, V_e_, V_p_, iAUC, and ADC maps) were fused with the structural MRI (T1CE or T_2_WI) in the workstation. Then, three manual circular-like ROIs among 20–40 mm^2^ were drawn by two experienced neuroradiologists (Z.Y.Z. and M.P.Y., with 3 and 8 years of experience in neoro-oncology imaging, respectively) on the significantly enhanced solid area of the tumor on T_1_CE (enhancing lesion), or the hyperintense region on T_2_WI in the central area of the tumor (non-enhancing lesion), at the axial slice with the maximum tumor area. The whole process avoided the peritumoral edema, hemorrhage, cysts, necrosis, calcification, and large vessels (21). The consistency of the MRI quantitative parameters averaging intensity above 3 ROIs from two neuro-radiologists was evaluated with the Intraclass Correlation Test. Under the condition of good consistency, the data extracted by the neuroradiologists with longer practice time prevailed. For the automatic way, the tumor core segmented by the neural network was consider as the ROI for calculating DCE perfusion parameters and ADC. Then, we compared the MRI quantitative parameters (K^trans^, K_ep_, V_e_, V_p_, iAUC, and ADC), extracted by manual delineation and automatic segmentation, and clinical index (age) between IDH1-mut and IDH1-wt groups. Parameters with statistically significant differences between groups were selected for input the following prediction models.

### Radiomics feature extraction

We used uAI Research Portal (Shanghai United Imaging Intelligence, Co., Ltd.) (41) to extract features from 3D-ROI of automatic segmentation. The uAI Research Portal is a clinical research platform and implemented by Python programming language (version 3.12.1, https://www.python.org). The widely used package-PyRadiomics (https://pyradiomics.readthedocs.io/en/latest/index.html) was embedded into this platform. The pre-processing of feature extraction included bias field correction, skull stripping, resampling, intensity normalization, and feature normalization. Specifically, the MRI images were resampled to the same spatial resolution, 1× 1 × 1 mm^3^, and the intensity values of each image were linearly normalized into the range [−1, 1]. The Radiomics features were computed from 3D-ROIs based on PyRadiomics. A total of 2,552 features were extracted based on conventional MR images (T_1_CE, T_2_W, and ADC), and 14,220 features were extracted based on DCE parameter images (K^trans^, K_ep_, V_e_, V_p_, and iAUC).

### Model construction and evaluation

The basic setup for the model was a pipeline consisting of four components as follows: a standardizer using Z-score normalization, feature selection using selection operator (LASSO) and the maximum correlation and minimum redundancy method, oversampling using the synthetic minority oversampling technique (SMOTE), and a classifier using logistic regression. The Z-score normalization algorithm was first used to remove the limitations imposed by the units of each feature. Subsequently, the dimension of the features was reduced by lasso regression analysis, then the most relevant features were selected by the maximum correlation and minimum redundancy method. Finally, 7 features were selected from conventional MR Radiomic features (T_1_CE, T_2_W, and ADC), 5 features were selected from DCE Radiomic features (K^trans^, K_ep_, V_e_, V_p_, and iAUC), and 9 features were selected from the combination of the abovementioned 12 two kinds of Radiomic features.

**Figure 3:**
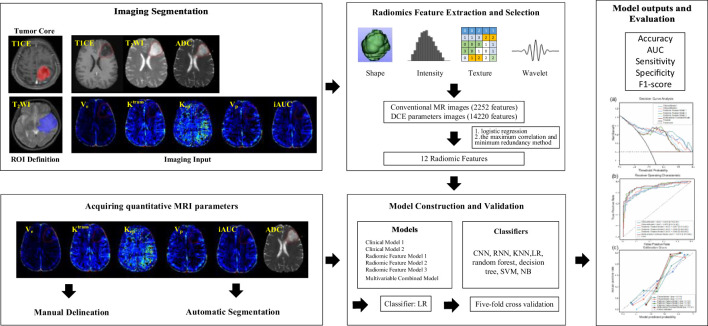
A study flow chart for predicting IDH1 mutation in gliomas. Firstly, Region of interest (ROIs) covering the tumor core was delineated according to T1CE or T_2_WI. Secondly, 16472 Radiomic features were totally extracted, seven features from conventional MR Radiomic features (T1CE, T_2_WI, ADC) and five features from DCE Radiomic features (K_trans_, K_ep_, V_e_, V_p_, and iAUC) were selected among them. Thirdly, two kinds of methods, manual delineation and automatic segmentation, were tried to obtain quantitative MRI parameters. Those parameters with differences between IDH1-mut and IDH1-wt groups were selected to input the following prediction models. Lastly, six models were constructed, the best classifier was selected by trying different usual classifiers and five-fold cross-validation was applied for validation. We compared the classification performance and selected the promising one among these six prediction models.

In our research, we used six kinds of different model inputs for data training, including: (a) Clinical Model 1: age, six MRI quantitative parameters obtained by manual extraction; (b) Clinical Model 2: age, six MRI quantitative parameters obtained by automatic extraction; (c) Radiomic Feature Model 1: 7 features selected from conventional MR Radiomic features; (d) Radiomic Feature Model 2: 5 features selected from DCE Radiomic features; (e) Radiomic Feature Model 3: 9 features selected from the above 12 selected Radiomic features; (f) Multivariable Combined Model: 11 features selected from multi-modality features (the above 9 twice-selected Radiomic features, age, and six MRI quantitative parameters by automatic extraction). For maximizing Radiomics algorithm’s discrimination, convolutional neural networks (CNN), recurrent neural network (RNN), K near neighbor (KNN), logistic regression (LR), random forest, decision tree, support vector machine (SVM), and Naive Bayes (NB) were implemented for model construction, respectively. Fivefold cross-validation was applied for validation. By trying different classifiers, we finally chose logistic regression according to a comparison of the classification results of the different models. The study workflow is depicted in **Figure 3**.

### Statistical analysis

Python (version 3.12.1, https://www.python.org). was used for data analyses and visualization in this study. Categorical variables were subjected to Chi-square testing, while continuous variables were analyzed using either the independent samples t-test or the Mann–Whitney U test, depending on their distribution characteristics. The intraclass correlation coefficient (ICC) was used to determine the intraobserver and interobserver agreement of ADC and DCE parameters. One‐way analysis of variance (ANOVA) and Bonferroni *post‐hoc* tests were used to test the differences in age and MRI quantitative parameters among groups. Values of p less than 0.05 indicated statistical significance. The diagnostic performance of the classification model was measured in terms of accuracy, area under the receiver operating characteristic curve (AUC), using the “PRROC” R package. The 95% confidence interval (CI) of the AUC was calculated from 2,000 iterations of bootstrapping with the predicted probabilities from the models. The probability threshold for the accuracy calculation was set to 0.5. Thus, a predicted probability of ≥0.5 was classified as an IDH mutation, and other values were classified as IDH wild type. To maximize the recognition rate of the Radiomics algorithm, multiple methods were used to construct models, respectively, such as the Convolutional Neural Network (CNN) and Logistic Regression (LR). The optimal cutoff was determined using Youden’s index. AUC comparisons were performed between the various models using Delong’s method. We mainly referred to the AUC value to compare the prediction performance of different common classifiers. Finally, we selected LR as the final classifier to compare the performance of different model inputs. Fivefold cross-validation was adopted to ensure the robustness and reliability of these models.

## Results

### Clinical characteristics

The baseline demographics and clinical characteristics of the study participants are summarized in **Table 1**. One hundred and fifty-two patients (55.8 ± 12.0 years; 79 females and 73 males) were included in this retrospective research, including 64 IDH-mutant (IDH1-mut) and 88 IDH1-wild type (IDH1-wt). The study participants were divided into two groups based on IDH1 gene status. Age was statistically significant between groups. For manual delineation, ADC value, K^trans^, K_ep_, V_e_, V_p_, and iAUC were statistically significant between groups. As for automatic segmentation, K^trans^, K_ep_, V_e_, V_p_, and iAUC were statistically significant between groups.

**Table 1:** Clinical features of patients in the IDH-mut and IDH1-wild groups.

Variables	IDH1 status		p-Value^*^
	IDH1-wt type	IDH1-mut	
Age (years)	60.88 ± 8.70	48.81 ± 12.46	< 0.001
Gender			0.225
Female, no. (%)	38 (43.2)	34 (53.1)	
Male, no. (%)	50 (56.8)	30 (46.9)	
Histology			NA
Glioblastoma, IDH-wild, no. (%)	88 (100)	0 (0)	
Astrocytoma, IDH-mut, no. (%)	0 (0)	45 (70)	
Oligodendroglioma, IDH-mut, no. (%)	0 (0)	19 (30)	
Grade			NA
G2, no. (%)	0 (0)	32 (50)	
G3, no. (%)	0 (0)	16 (25)	
G4, no. (%)	88 (100)	16 (25)	
Automatic segmentation			
ADC_0,1000_ (mm^2^/s)	1,206.37 ± 224.08	1,229.56 ± 269.00	0.564
K^trans^ (/min/1,000)	68.82 (49.42, 93.15)	31.99 (13.53, 52.93)	<0.001
K_ep_ (/min/1,000)	338.15 (279.00, 422.31)	572.04 (355.92, 1433.20)	<0.001
V_e_ (/100)	238.23 (171.37, 353.27)	68.32 (17.36, 164.70)	<0.001
V_p_ (ml/100 g/1,000)	4.59 (2.47, 8.67)	2.85 (1.53, 6.25)	0.004
iAUC (minmmol/L/100)	20.99 (15.42, 29.60)	8.24 (3.36, 15.38)	<0.001
Manual delineation			
ADC_0,1000_ (mm^2^/s)	1,009.30 ± 217.37	1,156.06 ± 283.11	0.001
K^trans^ (/min/1,000)	74.45 (48.55, 105.85)	29.05 (11.40, 55.23)	<0.001
K_ep_ (/min/1,000)	342.95 (235.40, 507.03)	729.20 (358.78, 1750.28)	<0.001
V_e_ (/100)	228.20 (139.70, 419.23)	48.30 (12.20, 216.33)	<0.001
V_p_ (ml/100 g/1,000)	6.30 (1.65, 16.25)	2.95 (1.10, 6.08)	0.005
iAUC (minmmol/L/100)	25.70 (19.13, 47.28)	8.30 (3.43, 23.58)	< 0.001

Data are presented as mean ± standard deviation, number with percentage in parentheses, or median with interquartile range in parentheses. *Calculated using chi-square test for categorical variables, either independent t test or Mann–Whitney U test for continuous variables depending on their normality distribution. G2, grade 2; G3, grade 3; G4, grade 4; ADC_0,1000_, apparent diffusion coefficient, calculated based on b0 and b1000; K^trans^, volume transfer constant; K_ep_, rate constant between the extravascular extracellular space and blood plasma; V_e_, volume of extravascular/extracellular space per unit volume of tissue; V_p_, fractional blood plasma volume; iAUC, initial area under the curve.

### Radiomic feature extraction and feature selection

A total of 2,552 features were extracted based on conventional MR images (T_1_CE, T_2_W, and ADC), and 14,220 features were extracted based on DCE parameter images (K^trans^, K_ep_, V_e_, V_p_, and iAUC). After applying logistic regression and the maximum correlation and minimum redundancy method, seven key Radiomic features were selected from conventional MR Radiomic features, and five features were selected from DCE Radiomic features.

### Model validation, evaluation, and comparison

**Figure 4:**
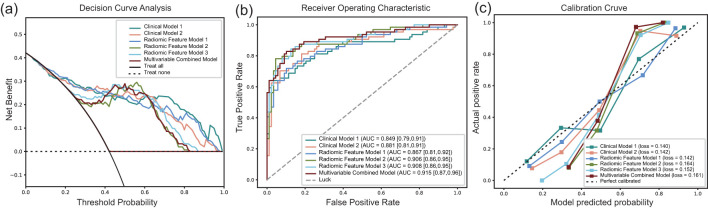
Evaluation of the different diagnosis model. **(a)** Decision curve analysis for six diagnosis models, the x-axis represents the threshold probability, and the y-axis measures the net benefit. **(b)** the ROC curve of different diagnosis model in the validation cohort. **(c)** the calibration curves for assessment of the clinical diagnosis models.

We mainly referred to the AUC value to compare the prediction performance among different usual classifiers. Finally, we selected LR as the final classifier to compare the performance of different model inputs. Five-old cross-validation was applied to ensure the robustness and reliability of these models. The results showed that the AUCs in the validation cohort of Clinical models 1–2, Radiomic feature models 1–3, and Multivariable Combined Model were 0.849 (95% CI: 0.79, 0.91), 0.881 (95% CI: 0.81, 0.91), 0.867 (95% CI: 0.81, 0.92), 0.906 (95% CI: 0.86, 0.95), 0.908 (95% CI: 0.86, 0.95), and 0.915 (95% CI: 0.87, 0.96), respectively. The accuracy in the validation cohort of the above models were 0.803, 0.849, 0.803, 0.836, 0.855 and 0.862, respectively. More details are summarized in **Table 2**; **Figure 4**. The Multivariable Combined Model showed the best classification performance. There was no significant difference between Clinical Model 1 and Clinical Model 2, Radiomic Feature Model 2 and Radiomic Feature Model 1 in the validation cohort (DeLong p > 0.05), whereas Radiomic Feature Model 3 performed better than Radiomic Feature Model 1 (DeLong p < 0.05). Multivariable Combined Model performed better than Clinical Model 1 and Radiomic Feature Model 1 (DeLong p all < 0.05). More details are summarized in **Supplementary Table S2**.

**Table 2:** The classification performance of the different diagnostic models.

Models	Features (no.)	Cohort	AUC	ACC	SEN	SPE	F1-score
Clinical Model 1	7	Training	0.871	0.829	0.719	0.909	0.780
		Validation	0.849	0.803	0.688	0.886	0.739
Clinical Model 2	7	Training	0.893	0.847	0.734	0.929	0.802
		Validation	0.881	0.849	0.703	0.932	0.790
Radiomic Feature Model 1	7	Training	0.884	0.816	0.734	0.875	0.770
		Validation	0.867	0.803	0.719	0.864	0.748
Radiomic Feature Model 2	5	Training	0.908	0.842	0.809	0.867	0.812
		Validation	0.906	0.836	0.797	0.864	0.802
Radiomic Feature Model 3	9	Training	0.914	0.862	0.801	0.906	0.830
		Validation	0.908	0.855	0.781	0.909	0.814
Multivariable Combined Model	11	Training	0.917	0.857	0.789	0.906	0.823
		Validation	0.915	0.862	0.797	0.909	0.824

AUC, area under the curve; ACC, accuracy; SEN, sensitivity; SPE, specificity.

## Discussion

We developed and validated a multiparametric prognostic model to predict IDH mutation status in glioma using MR Radiomics features combined with clinical factor (age) and MRI quantitative parameters (DCE and ADC), which showed improved performance over the model using only conventional MRI or mono-modality radiomic features. Compared with the independent Radiomics and Clinical Models, the Multivariable Combined Model could be the most promising, and in the current study, its advantages are deserved for attention. First, we provided a binary classifier to preoperatively predict IDH mutation of glioma with satisfactory performance. Second, our model made full use of DCE quantitative parameters and their Radiomics features to explore the value of MRI perfusion imaging in predicting IDH-mut glioma. Third, we compared the differences between MRI quantitative parameters extracted by manual 2D-ROI and automatic 3D- ROI.

The IDH1/2 mutation plays a crucial role in glioma in glioma diagnosis, treatment, and prognosis (42, 43). Previous studies have shown that IDH1/2 gene mutation can cause α-ketoglutaric acid reduction and 2-hydroxyglutaric acid increase, indirectly affect angiogenesis, hypoxia stress, cell maturation and differentiation, and other physiological and pathological processes, and interfere with the expression of vascular endothelial growth factor and other tumor-related genes (6, 44). Lusien et al. (45) concluded that gliomas with different IDH genotypes had unique vascular gene expression patterns related to vascular remodeling. Yue Hu et al. (46) showed that IDH1-wt gliomas expressed significantly higher vascular endothelial growth factor (VEGF) expression and perfusion metrics in contrast to IDH-mut gliomas. Multiple studies have shown that perfusion features obtained by perfusion-weighted MRI could predict glioma survival/progression and the critical tumor characteristics of glioma such as genetic mutations (47, 48). Yue Hu et al. (46) demonstrated that histogram analysis of DCE-MRI could non-invasively predict IDH mutation. In addition, histogram DCE-MRI also showed good diagnostic performance in predicting O6-methylguanine-DNA methyltransferase (MGMT), telomere reverse transcriptase (TERT), and evaluating the prognosis of glioma (49). These insights in phenotype and genotype account for the application of perfusion MRI to predict IDH mutation. It is also valuable and persuasive to use multi-modality Radiomic features including DCE to predict IDH genotype.

Zhang et al. found that the histogram DCE-MRI demonstrated good diagnostic performance in identifying IDH1 mutation (49). We found that the values of K^trans^, V_e_, and iAUC in IDH wild-type gliomas were higher than those of IDH mutant gliomas, and the values of K_ep_ were lower than those of IDH mutant gliomas. Furthermore, the difference between groups of ADC values obtained by automatic extraction was inconsistent with manual extraction, which should be related to the heterogeneity of gliomas and the delineated area of ROI. Ignoring this difference, we included six quantitative MRI parameters and age to predict glioma IDH mutation in two clinical models. We hope to explore the effect of MRI quantitative parameters obtained by two different ROI extraction methods on the prediction of IDH mutation in clinical models. We found that the AUC of Clinical Model 2 (automatic extraction) was higher than that of Clinical Model 1 (manual extraction) in the testing cohort (DeLong p > 0.05). We consider that the influence of different ROI sketching methods on quantitative MRI studies could be ignored.

In the following research, we constructed three Radiomic feature models and one Multivariable Combined Model. We found that the classification performance of the Radiomic feature model of DCE quantitative parameters was higher than the model of traditional MRI image features (DeLong p > 0.05), and lower than the combination model of these two kinds of Radiomic features (DeLong p > 0.05). Among these models, the Multivariable Combined Model showed the best classification performance. We considered the quantitative MRI parameters may not have obvious influence on the classification results of the Multivariable Combined Model, and the difference between the model of Radiomic features and the model of quantitative MRI parameters was not significant (p > 0.05). The AUC of the Multivariable Combined Model in testing cohort was 0.915 and the accuracy was 0.862, which deserved people’s concern while comparing with previous researches. Wang et al. found the Radiomics model based on DCE-MRI and DWI had a considerable effect on the evaluation of IDH1 mutation (50), and the AUC and accuracy of the combined model in testing cohort was 0.909,0.833 separately. Hitherto, there are no studies focusing on the estimation of IDH1 mutation using the Radiomics analysis of DCE-MRI and quantitative MRI parameters of DCE. In addition, 3D-ROI was also used in this study to automatically segment the entire lesion and extract DCE quantitative parameters, which was not been tried in previous studies. Our study demonstrated that the LR model based on multivariable combined parameters showed good diagnostic performance in estimating IDH1 mutation in gliomas.

The present study has several limitations. First, the study was retrospective, limited radiomic features restricted further improvement of the model performance. Second, the radiomic model may not perform well in multi-centered and future applications because to the sensitivity of radiomic characteristics to parameters and systems. Our next research topic is multicenter radiomic, whose harmonization can improve the generalizability of the model. Third, the time-consuming, multi-staged workflow discourages the application of the Multivariable Combined Model in clinical practice. In conclusion, we developed and validated a multiparametric prognostic model to predict IDH mutation status in glioma using MR Radiomics features combined with clinical factor and MRI quantitative parameters, which showed improved performance over the model using only conventional MRI or Radiomic features.

## Data Availability

The raw data supporting the conclusions of this article will be made available by the authors, without undue reservation.

## References

[1] Louis DN Perry A Wesseling P Brat DJ Cree IA Figarella-Branger D. The 2021 WHO classification of tumors of the central nervous system: a summary. Neuro-Oncology. (2021) 23:1231–51. doi: 10.1093/neuonc/noab106 PMC8328013 34185076

[2] Iorgulescu JB Sun C Neff C Cioffi G Gutierrez C Kruchko C. Molecular biomarker-defined brain tumors: Epidemiology, validity, and completeness in the United States. Neuro-Oncology. (2022) 24:1989–2000. doi: 10.1093/neuonc/noac113 35460555 PMC9629432

[3] Miller JJ Gonzalez Castro LN McBrayer S Weller M Cloughesy T Portnow J. Isocitrate dehydrogenase (IDH) mutant gliomas: A Society for Neuro-Oncology (SNO) consensus review on diagnosis, management, and future directions. Neuro Oncol. (2023) 25:4–25. doi: 10.1093/neuonc/noac207 36239925 PMC9825337

[4] Kong Z Jiang C Zhang Y Liu S Liu D Liu Z. Thin-slice magnetic resonance imaging-based radiomics signature predicts chromosomal 1p/19q co-deletion status in grade II and III gliomas. Front Neurol. (2020) 11:551771. doi: 10.3389/fneur.2020.551771 33192984 PMC7642873

[5] Berger TR Wen PY Lang-Orsini M Chukwueke UN. World health organization 2021 classification of central nervous system tumors and implications for therapy for adult-type gliomas: A review. JAMA Oncol. (2022) 8:1493–501. doi: 10.1001/jamaoncol.2022.2844 36006639

[6] Harding JJ Lowery MA Shih AH Schvartzman JM Hou S Famulare C. Isoform switching as a mechanism of acquired resistance to mutant isocitrate dehydrogenase inhibition. Cancer Discovery. (2018) 8:1540–7. doi: 10.1158/2159-8290.CD-18-0877 PMC6699636 30355724

[7] Hartmann C Hentschel B Wick W Capper D Felsberg J Simon M. Patients with IDH1 wild type anaplastic astrocytomas exhibit worse prognosis than IDH1-mutated glioblastomas, and IDH1 mutation status accounts for the unfavorable prognostic effect of higher age: implications for classification of gliomas. Acta Neuropathol. (2010) 120:707–18. doi: 10.1007/s00401-010-0781-z 21088844

[8] Beiko J Suki D Hess KR Fox BD Cheung V Cabral M. IDH1 mutant Malignant astrocytomas are more amenable to surgical resection and have a survival benefit associated with maximal surgical resection. Neuro Oncol. (2014) 16:81–91. doi: 10.1093/neuonc/not159 24305719 PMC3870823

[9] Ahmeti H Kiese D Freitag-Wolf S Kalab M Röcken C Jansen O. IDH1 mutation is associated with improved resection rates, progression-free survival and overall survival in patients with anaplastic astrocytomas. J Neurooncol. (2024) 169:423–35. doi: 10.1007/s11060-024-04743-x 38909340

[10] Mellinghoff IK Lu M Wen PY Taylor JW Maher EA Arrillaga-Romany I. Vorasidenib and ivosidenib in IDH1-mutant low-grade glioma: a randomized, perioperative phase 1 trial. Nat Med. (2023) 29:615–22. doi: 10.1038/s41591-022-02141-2 PMC10313524 36823302

[11] Mellinghoff IK Penas-Prado M Peters KB Burris HA Maher EA Janku F. Vorasidenib, a dual inhibitor of mutant IDH1/2, in recurrent or progressive glioma; results of a first-in-human phase I trial. Clin Cancer Res. (2021) 27:4491–9. doi: 10.1158/1078-0432.CCR-21-0611 PMC8364866 34078652

[12] Mellinghoff IK van den Bent MJ Blumenthal DT Touat M Peters KB Clarke J. Vorasidenib in IDH1- or IDH2-mutant low-grade glioma. N Engl J Med. (2023) 389:589–601. doi: 10.1056/NEJMoa2304194 37272516 PMC11445763

[13] Ahmad O Ahmad T Pfister SM. IDH mutation, glioma immunogenicity, and therapeutic challenge of primary mismatch repair deficient IDH-mutant astrocytoma PMMRDIA: a systematic review. Mol Oncol. (2024) 18:2822–41. doi: 10.1002/1878-0261.13598 PMC11619801 38339779

[14] Yan D Li W Liu Q Yang K. Advances in immune microenvironment and immunotherapy of isocitrate dehydrogenase mutated glioma. Front Immunol. (2022) 13:914618. doi: 10.3389/fimmu.2022.914618 35769466 PMC9234270

[15] Yang K Wu Z Zhang H Zhang N Wu W Wang Z. Glioma targeted therapy: insight into future of molecular approaches. Mol Cancer. (2022) 21:39. doi: 10.1186/s12943-022-01513-z 35135556 PMC8822752

[16] Haubold J Hosch R Parmar V Glas M Guberina N Catalano OA. Fully automated MR based virtual biopsy of cerebral gliomas. Cancers. (2021) 13:6186. doi: 10.3390/cancers13246186 34944806 PMC8699054

[17] Sha Y Yan Q Tan Y Wang X Zhang H Yang G. Prediction of the molecular subtype of IDH mutation combined with MGMT promoter methylation in gliomas via radiomics based on preoperative MRI. Cancers. (2023) 15:1440. doi: 10.3390/cancers15051440 36900232 PMC10001198

[18] Wang Z Guan F Duan W Guo Y Pei D Qiu Y. Diffusion tensor imaging-based machine learning for IDH wild-type glioblastoma stratification to reveal the biological underpinning of radiomic features. CNS Neurosci Ther. (2023) 29:3339–50. doi: 10.1111/cns.14263 PMC10580329 37222229

[19] Yuan Y Yu Y Chang J Chu Y-H Yu W Hsu Y-C. Convolutional neural network to predict IDH mutation status in glioma from chemical exchange saturation transfer imaging at 7 Tesla. Front Oncol. (2023) 13:1134626. doi: 10.3389/fonc.2023.1134626 37223677 PMC10200907

[20] Pei D Guan F Hong X Liu Z Wang W Qiu Y. Radiomic features from dynamic susceptibility contrast perfusion-weighted imaging improve the three-class prediction of molecular subtypes in patients with adult diffuse gliomas. Eur Radiol. (2023) 33:3455–66. doi: 10.1007/s00330-023-09459-6 36853347

[21] Yang H Zhu Z Long C Niu F Zhou J Chen S. Quantitative and qualitative parameters of DCE-MRI predict CDKN2A/B homozygous deletion in gliomas. Acad Radiol. (2024) 31:3355–65. doi: 10.1016/j.acra.2024.02.017 38443208

[22] Zhu Z Shen J Liang X Zhou J Liang J Ni L. Radiomics for predicting grades, isocitrate dehydrogenase mutation, and oxygen 6-methylguanine-DNA methyltransferase promoter methylation of adult diffuse gliomas: combination of structural MRI, apparent diffusion coefficient, and susceptibility-weighted imaging. Quant Imaging Med Surg. (2024) 14:9276–89. doi: 10.21037/qims-24-1110 PMC11652054 39698654

[23] Hangel G Schmitz-Abecassis B Sollmann N Pinto J Arzanforoosh F Barkhof F. Advanced MR techniques for preoperative glioma characterization: part 2. J magnetic resonance imaging: JMRI. (2023) 57:1676–95. doi: 10.1002/jmri.28663 PMC10947037 36912262

[24] Nalepa J Ribalta Lorenzo P Marcinkiewicz M Bobek-Billewicz B Wawrzyniak P Walczak M. Fully-automated deep learning-powered system for DCE-MRI analysis of brain tumors. Artif Intell Med. (2020) 102:101769. doi: 10.1016/j.artmed.2019.101769 31980106

[25] Liang J Liu D Gao P Zhang D Chen H Shi C. Diagnostic values of DCE-MRI and DSC-MRI for differentiation between high-grade and low-grade gliomas: A comprehensive meta-analysis. Acad Radiol. (2018) 25:338–48. doi: 10.1016/j.acra.2017.10.001 29223713

[26] Parvaze S Bhattacharjee R Singh A Ahlawat S Patir R Vaishya S. Radiomics-based evaluation and possible characterization of dynamic contrast enhanced (DCE) perfusion derived different sub-regions of Glioblastoma. Eur J Radiol. (2023) 159:110655. doi: 10.1016/j.ejrad.2022.110655 36577183

[27] Wang S Sun K Wang L Qu L Yan F Wang Q. Breast tumor segmentation in DCE-MRI with tumor sensitive synthesis. IEEE Trans Neural Networks Learn Syst. (2023) 34:4990–5001. doi: 10.1109/TNNLS.2021.3129781 34874872

[28] Zhao X Liao Y Xie J He X Zhang S Wang G. BreastDM: A DCE-MRI dataset for breast tumor image segmentation and classification. Comput Biol Med. (2023) 164:107255. doi: 10.1016/j.compbiomed.2023.107255 37499296

[29] Tavakoli AA Hielscher T Badura P Görtz M Kuder TA Gnirs R. Contribution of dynamic contrast-enhanced and diffusion MRI to PI-RADS for detecting clinically significant prostate cancer. Radiology. (2023) 306:186–99. doi: 10.1148/radiol.212692 35972360

[30] Breit HC Block TK Winkel DJ Gehweiler JE Glessgen CG Seifert H. Revisiting DCE-MRI: classification of prostate tissue using descriptive signal enhancement features derived from DCE-MRI acquisition with high spatiotemporal resolution. Invest Radiol. (2021) 56:553–62. doi: 10.1097/RLI.0000000000000772 PMC8373655 33660631

[31] Bonada M Rossi LF Carone G Panico F Cofano F Fiaschi P. Deep learning for MRI segmentation and molecular subtyping in glioblastoma: critical aspects from an emerging field. Biomedicines. (2024) 12:1878. doi: 10.3390/biomedicines12081878 39200342 PMC11352020

[32] Bianconi A Rossi LF Bonada M Zeppa P Nico E De Marco R. Deep learning-based algorithm for postoperative glioblastoma MRI segmentation: a promising new tool for tumor burden assessment. Brain Inform. (2023) 10:26. doi: 10.1186/s40708-023-00207-6 37801128 PMC10558414

[33] van der Voort SR Incekara F Wijnenga MMJ Kapsas G Gahrmann R Schouten JW. Combined molecular subtyping, grading, and segmentation of glioma using multi-task deep learning. Neuro-oncol. (2023) 25:279–89. doi: 10.1093/neuonc/noac166 PMC9925710 35788352

[34] Luo J Pan M Mo K Mao Y Zou D. Emerging role of artificial intelligence in diagnosis, classification and clinical management of glioma. Semin Cancer Biol. (2023) 91:110–23. doi: 10.1016/j.semcancer.2023.03.006 36907387

[35] Shi F Hu W Wu J Han M Wang J Zhang W. Deep learning empowered volume delineation of whole-body organs-at-risk for accelerated radiotherapy. Nat Commun. (2022) 13:6566. doi: 10.1038/s41467-022-34257-x 36323677 PMC9630370

[36] Milletari F Navab N Ahmadi S-A. (2016). V-net: fully convolutional neural networks for volumetric medical image segmentation, in: 2016 Fourth International Conference on 3D Vision (3DV), California, 2016. the USA: IEEE. pp. 565–71.

[37] Chen S Song D Chen L Guo T Jiang B Liu A. Artificial intelligence-based non-invasive tumor segmentation, grade stratification and prognosis prediction for clear-cell renal-cell carcinoma. Precis Clin Med. (2023) 6:pbad019. doi: 10.1093/pcmedi/pbad019 38025974 PMC10680020

[38] Zhu W Huang H Zhou Y Shi F Shen H Chen R. Automatic segmentation of white matter hyperintensities in routine clinical brain MRI by 2D VB-Net: A large-scale study. Front Aging Neurosci. (2022) 14:915009. doi: 10.3389/fnagi.2022.915009 35966772 PMC9372352

[39] Yang C Zhou Q Li M Xu L Zeng Y Liu J. MRI-based automatic identification and segmentation of extrahepatic cholangiocarcinoma using deep learning network. BMC Cancer. (2023) 23:1089. doi: 10.1186/s12885-023-11575-x 37950207 PMC10636947

[40] Tseng C-H Jaspers J Romero AM Wielopolski P Smits M van Osch MJP. Improved reliability of perfusion estimation in dynamic susceptibility contrast MRI by using the arterial input function from dynamic contrast enhanced MRI. Nmr BioMed. (2024) 37:e5038. doi: 10.1002/nbm.5038 37712359

[41] Wu J Xia Y Wang X Wei Y Liu A Innanje A. uRP: An integrated research platform for one-stop analysis of medical images. Front Radiol. (2023) 3:1153784. doi: 10.3389/fradi.2023.1153784 37492386 PMC10365282

[42] Śledzińska P Bebyn MG Furtak J Kowalewski J Lewandowska MA. Prognostic and predictive biomarkers in gliomas. Int J Mol Sci. (2021) 22:10373. doi: 10.3390/ijms221910373 34638714 PMC8508830

[43] Han S Liu Y Cai SJ Qian M Ding J Larion M. IDH mutation in glioma: molecular mechanisms and potential therapeutic targets. Br J Cancer. (2020) 122:1580–9. doi: 10.1038/s41416-020-0814-x PMC7250901 32291392

[44] Figueroa ME Abdel-Wahab O Lu C Ward PS Patel J Shih A. Leukemic IDH1 and IDH2 mutations result in a hypermethylation phenotype, disrupt TET2 function, and impair hematopoietic differentiation. Cancer Cell. (2010) 18:553–67. doi: 10.1016/j.ccr.2010.11.015 PMC4105845 21130701

[45] van Santwijk L Kouwenberg V Meijer F Smits M Henssen D. A systematic review and meta-analysis on the differentiation of glioma grade and mutational status by use of perfusion-based magnetic resonance imaging. Insights into Imaging. (2022) 13:102. doi: 10.1186/s13244-022-01230-7 35670981 PMC9174367

[46] Hu Y Chen Y Wang J Kang JJ Shen DD Jia ZZ. Non-invasive estimation of glioma IDH1 mutation and VEGF expression by histogram analysis of dynamic contrast-enhanced MRI. Front Oncol. (2020) 10:593102. doi: 10.3389/fonc.2020.593102 33425744 PMC7793903

[47] Choi KS Choi SH Jeong B. Prediction of IDH genotype in gliomas with dynamic susceptibility contrast perfusion MR imaging using an explainable recurrent neural network. Neuro Oncol. (2019) 21:1197–209. doi: 10.1093/neuonc/noz095 PMC7594560 31127834

[48] Kim M Jung SY Park JE Jo Y Park SY Nam SJ. Diffusion- and perfusion-weighted MRI radiomics model may predict isocitrate dehydrogenase (IDH) mutation and tumor aggressiveness in diffuse lower grade glioma. Eur Radiol. (2020) 30:2142–51. doi: 10.1007/s00330-019-06548-3 31828414

[49] Zhang H-W Lyu G-W He W-J Lei Y Lin F Wang M-Z. DSC and DCE histogram analyses of glioma biomarkers, including IDH, MGMT, and TERT, on differentiation and survival. Acad Radiol. (2020) 27:e263–71. doi: 10.1016/j.acra.2019.12.010 31983532

[50] Wang J Hu Y Zhou X Bao S Chen Y Ge M. A radiomics model based on DCE-MRI and DWI may improve the prediction of estimating IDH1 mutation and angiogenesis in gliomas. Eur J Radiol. (2022) 147:110141. doi: 10.1016/j.ejrad.2021.110141 34995947

