# Supplementary material for: Integrating quantitative DCE-MRI parameters and radiomic features for improved IDH mutation prediction in gliomas
Source: Front Oncol. 2025 Mar 11;15:1530144. doi: 10.3389/fonc.2025.1530144 (PMC11932857; doi:10.3389/fonc.2025.1530144)
Supplement: Supplementary file 1 [file DataSheet1.docx]

**Supplemental Tables**

**Table S1** The protocol details of the conventional MRI sequences

| Sequence | FOV (mm) | TR (ms) | TE (ms) | Matrix | Scan Time (s) |
| --- | --- | --- | --- | --- | --- |
| DWI | 1.67 × 1.67 × 6 | 1800 | 86 | 144 × 138 | 39.9 |
| T_1_WI | 1.02 × 0.76 × 6 | 230 | 4.49 | 288 × 216 | 34.6 |
| T_2_WI | 1 × 1 × 1 | 1500 | 369.6 | 256 × 272 | 117 |
| T_1_CE | 1 × 1 × 1 | 7.2 | 3.1 | 256 × 270 | 154 |

FOV: Field of View; TR: Repetition Time; TE: Echo Time.

**Table S2** The results of DeLong Test between different prediction models in validation cohort

| Model 1 | AUC | Model 2 | AUC | P |
| --- | --- | --- | --- | --- |
| Clinical Model 1 | 0.849 | Clinical Model 2 | 0.881 | 0.147 |
| Clinical Model 1 | 0.849 | Radiomic Feature Model 1 | 0.867 | 0.614 |
| Clinical Model 1 | 0.849 | Radiomic Feature Model 2 | 0.906 | 0.072 |
| Clinical Model 1 | 0.849 | Radiomic Feature Model 3 | 0.908 | 0.065 |
| Clinical Model 1 | 0.849 | Multivariable Combined Model | 0.910 | 0.027^*^ |
| Clinical Model 2 | 0.881 | Radiomic Feature Model 1 | 0.867 | 0.565 |
| Clinical Model 2 | 0.881 | Radiomic Feature Model 2 | 0.906 | 0.293 |
| Clinical Model 2 | 0.881 | Radiomic Feature Model 3 | 0.908 | 0.235 |
| Clinical Model 2 | 0.881 | Multivariable Combined Model | 0.910 | 0.053 |
| Radiomic Feature Model 1 | 0.867 | Radiomic Feature Model 2 | 0.906 | 0.108 |
| Radiomic Feature Model 1 | 0.867 | Radiomic Feature Model 3 | 0.908 | 0.026^*^ |
| Radiomic Feature Model 1 | 0.867 | Multivariable Combined Model | 0.910 | 0.019^*^ |
| Radiomic Feature Model 2 | 0.906 | Radiomic Feature Model 3 | 0.908 | 0.817 |
| Radiomic Feature Model 2 | 0.906 | Multivariable Combined Model | 0.910 | 0.731 |
| Radiomic Feature Model 3 | 0.908 | Multivariable Combined Model | 0.910 | 0.779 |

AUC: area under the receiver operating characteristic curve; *: p < 0.05, the difference was statistically significant.
